# COVID-19 Vaccines: Current Understanding on Immunogenicity, Safety, and Further Considerations

**DOI:** 10.3389/fimmu.2021.669339

**Published:** 2021-04-12

**Authors:** Qian He, Qunying Mao, Jialu Zhang, Lianlian Bian, Fan Gao, Junzhi Wang, Miao Xu, Zhenglun Liang

**Affiliations:** National Institutes for Food and Drug Control, Beijing, China

**Keywords:** COVID-19 vaccines, immunogenicity, safety profile, efficacy, reference standard

## Abstract

The world has entered the second wave of the COVID-19 pandemic, and its intensity is significantly higher than that of the first wave of early 2020. Many countries or regions have been forced to start the second round of lockdowns. To respond rapidly to this global pandemic, dozens of COVID-19 vaccine candidates have been developed and many are undergoing clinical testing. Evaluating and defining effective vaccine candidates for human use is crucial for prioritizing vaccination programs against COVID-19. In this review, we have summarized and analyzed the efficacy, immunogenicity and safety data from clinical reports on different COVID-19 vaccines. We discuss the various guidelines laid out for the development of vaccines and the importance of biological standards for comparing the performance of vaccines. Lastly, we highlight the key remaining challenges, possible strategies for addressing them and the expected improvements in the next generation of COVID-19 vaccines.

## Introduction

As of January 2021, the cumulative number of confirmed cases of COVID-19 worldwide has reached nearly 100 million, and the death toll has exceeded 2 million (1). The astounding speed of COVID-19 vaccine development as a counter-measure is unparalleled in the history of vaccine development (2). In addition, COVID-19 is the first disease where hundreds of institutions and companies are engaged in research for producing effective vaccines from multiple platforms established in-parallel. By the end of 2020, more than 60 vaccines entered the clinic trials, with 13 in Phase 3 clinical trials (3), among which mRNA vaccines (Pfizer-BioNTech, Moderna), recombinant adenovirus vectored vaccines (AstraZeneca, Cansino, Gamaleya, Johnson Pharm) and inactivated vaccines (Sinopharm, Sinovac), have made the fastest progress. As of the end of 2020, nine candidate vaccines have been authorized for human use in many countries (4–11). On the last day of 2020, the WHO authorized Pfizer vaccines for emergency use worldwide, opening the door to mass vaccination programs against COVID-19 (12). The approval of these vaccines provides a tool for global control of the COVID-19 epidemic. Never in the history of human vaccines have so many vaccines been developed and entered the clinical research in such a short period of time and obtained authorization for emergency use or conditional marketing.

An ideal vaccine should confer effective protection for a long time, possess a good safety profile, should be affordable and be easily accessible to all (13). It is difficult to develop vaccines with such attributes in a short time. Due to the unprecedented nature of the epidemic and the subsequent urgency shown in the research and development, the antigen construction and production processes of the COVID-19 vaccines developed for emergency use have not been fully optimized. How to scientifically dissect and evaluate the current COVID-19 vaccine candidates developed from different technical platforms, and then select vaccines suitable for their own country to contain the COVID-19 epidemic as quickly as possible, remains the most critical challenge faced by many countries across the world. Scientific evaluation of the COVID-19 vaccines is particularly important for regulatory agencies in various countries for (i) prioritization of safe and effective vaccines among a large number of vaccines under clinical trials (ii) aiding in deciding on an effective vaccine for non-inferiority trials to develop second-generation vaccines (14).

In this paper, we have reviewed and analyzed the clinical reports of different COVID-19 vaccines and have found that the currently developed COVID-19 vaccines differ significantly in their effectiveness and safety. Based on the results of the published studies, the vaccines could be improved by optimizing antigen design, screening adjuvants, testing different formulations, and exploring alternative vaccination regimens. Sequential immunization is likely to further improve the protective immune responses and safety profiles of the currently developed COVID-19 vaccines. The studies highlight the need to develop vaccines effective against newly emerging variant strains and with longer duration of immunity. The criteria and reference standards described by various regulatory bodies for COVID-19 vaccine evaluation are reviewed in this paper, which could be adopted and can be beneficial for vaccine development by developers as well as manufacturers.

## Features and Status of Different COVID-19 Vaccine Platforms

To quickly respond to the COVID-19 pandemic, a broad range of COVID-19 vaccine candidates have been investigated using various technologies and platforms including, nucleic acid (DNA, RNA) vaccines, viral vectored vaccines, inactivated vaccines, protein subunit and live attenuated vaccines (15, 16). The research status of currently developed COVD-19 vaccines can be checked at the WHO website (https://www.who.int/) and Clinical Trial website (https://clinicaltrials.gov/). Efforts have been primarily focused on targeting the viral cellular entry mechanism and various strategies on presenting the related antigens have been studied for the first wave of vaccines (17–21). The core ingredients of the current COVID-19 vaccines are mainly the Spike (S) protein (such as mRNA-1273, BNT162b2, Ad26.COV2.S, AZD1222, Ad5-nCoV, Sputnik V, NVX-Cov2373, SCB-2019, INO-4800, etc.) or Receptor Binding Domain (RBD) in S (such as ZF2001, BNT162b1, ARcoVax, etc.) in order to induce corresponding antibody to block the entry of SARS-CoV-2 into host cell. To improve the quality and quantity of vaccine-induced antibodies targeting the functionally relevant epitopes, strategies to stabilize the S protein in its pre-fusion conformation and enhance pre-fusion S protein expression have been adopted by vaccine developers (19).

### Nucleic Acid Vaccine

There are 7 mRNA-based COVID-19 vaccines and 10 DNA-based COVID-19 vaccines in clinical trials. In addition, 38 nucleic acid vector-based vaccines (23 mRNA vaccines, 15 DNA vaccines) are undergoing preclinical research (3). Among these, mRNA vaccines BNT162b2 (Pfizer-BioNTech) and mRNA-1273 (Moderna) are the first ones to have been approved for conditional marketing or emergency use by FDA, EMA and MHRA (4–6, 8, 10, 22). The speed of the development of mRNA vaccines was described as “lightning fast” by Nature journal (2). Both BNT162b2 (23) and mRNA-1273 (24) encode a full-length S protein, which is stabilized to pre-fusion conformation by two proline substitutions (K986 and V987) (S-2P) in cleavage site. The salient features of mRNA vaccines are: (i) Translation of mRNA occurs in the cytoplasm to avoid the risk of any genome integration. (ii) Both T cell and antibody responses are triggered. (iii) Rapid development and large-scale mass production can be achieved. Some of the drawbacks include the requirement of a delivery system or material for the entry of mRNA into the cell. In addition, mRNA is inherently unstable, requiring low temperature and ultra-low temperature storage and transportation. Such facilities are unlikely to be available in many developing countries, which could prevent the use of such effective vaccines in these countries (25).

DNA vaccine is another type of nucleic acid vaccine. The DNA vaccine INO-4800 developed by Inovio in the United States is making the fastest progress and is in phase 2 clinical trials. The strategy for INO-4800 included the insertion of the full-length sequence of the SARS-CoV-2 spike sequence into the pGX0001 plasmid vector for expressing S protein in host cells for eliciting specific immune responses. The advantages of this platform include: (i) S protein from multiple strains of SARS-CoV-2 can be cloned and screened simultaneously. (ii) Potent T cell and antibody-based immune responses can be induced; though the titers of antibodies induced are relatively low. (iii) The synthetic DNA vaccines are stable and can be stored at room temperature for 1 year or longer. However, there is a theoretical risk of gene integration while using DNA vaccines, but that has not been confirmed (26). Till January 2021, only Inovio has reported the immunogenicity results of the phase I clinical trials of its DNA vaccine. The sero-conversion rate of neutralizing antibodies reported was above 80% (27).

### Viral Vectored Vaccine

A variety of viral vectors like adenovirus, influenza, measles, ankara pox virus (MVA), and vesicular stomatitis virus (VSV) have been used in the development of COVID-19 vaccines. Amongst these, adenovirus vectored vaccines account for 1/3rd of the total number of vaccines being developed and have made the fastest progress (3). The characteristics of this vaccine platform include: (i) It mimics natural course of the infection to a certain extent, expressing antigens in the cell. Hence, strong humoral and cytotoxic immune responses are induced. (ii) The pre-existing or vaccine-elicited immune response against the viral vector limits its efficacy. (iii) There is a risk of gene integration, but it has not been confirmed. There are currently three vaccines of this type: “ Type 5 Adenovirus- vectored Ebola Vaccine “ (28), “VSV-Vectored Ebola Vaccine” (29) and “Yellow Fever Virus-Vectored Dengue Vaccine”, which have been conditionally approved for use in high-risk population groups. However, this type of vaccine has neither been used as a preventive vaccine in humans at a large scale nor tested for a long period before. Therefore, the efficacy and safety need to be continuously monitored.

At the end of 2020, there were four adenovirus vectored vaccines that entered phase 3 clinical trials, including AZD1222 (AstraZeneca) (30, 31), Ad5-nCoV(Cansino) (32), Sputnik V (Gamaleya) (33) and Ad26.COV2.S (Johnson Pharm) (34). All these four adenovirus-vectored COVID-19 vaccines were designed to express the full-length S protein. Amongst, Ad26.COV2.S encodes a stabilized S protein with furin cleavage site mutations and two proline substitutions (35). Among them, Sputnik V was registered on 11 August 2020 by the Russian Ministry of Health as Gam-COVID-Vac for human use (9), and AZD1222 was officially approved by MHRA for conditional marketing authorization in December 2020 (7). Prior exposure of the population to the vectors employed has a bearing on the induction of immune responses. For instance, the chimpanzee adenovirus ChAdOx1 used by AstraZeneca is characterized by low pre-existing immunity against the vector among the population (36); Furthermore, human adenovirus serotype 26 (Ad26) used by Johnson Pharm exhibits lower seroprevalence in humans and can bypass the pre-existing immunity against adenovirus serotype 5 (Ad5), making it an attractive vaccine vector alternative (37); Lastly, Sputnik V is a combined vectored vaccine based on Ad26 and Ad5, which theoretically combines the advantages of the two vectors and avoid pre-existing immunity against vaccine vector caused by homologous prime-boost. In addition to adenovirus, there are many other types of viral vectored vaccines undergoing clinical research. MERCK’s two COVID-19 vaccines, V590 and V591, are developed basing on VSV and measles virus, respectively, but were eventually discontinued for poor immunogenicity appeared in Phase 1 trial (38).

### Inactivated Vaccine

Inactivated vaccines are traditional and classic vaccine types. There are currently 10 inactivated vaccines in clinical trials of which Chinese companies account for 5 of them (3). The inactivated vaccines BBIBP-CorV and CoronaVac developed by Sinopharm and Sinovac, respectively, lead the pack and have already been approved for conditional marketing authorization in several countries by the end of 2020 (11). The BBV152, inactivated vaccine developed by Bharat Biotech in India, is the fifth inactivated vaccine that entered clinical trials (39), and has also been approved for emergency use in India. As one of the classic vaccine platforms, purified inactivated vaccine has been widely used to protect humans against rabies, polio, hepatitis B, influenza and other viruses. The safety profiles of these inactivated vaccines have been thoroughly tested and confirmed by mass immunization programs. A number of development and evaluation standards have been designed based on experience gained from the use of these vaccines. The manufacturing processes are mature for the large scale production of inactivated vaccines. Although, inactivated vaccines offer many advantages, they generally manifest moderate immunogenicity and require to be assisted by adjuvants and more than one dose of booster immunization. Moreover, specialized facilities like biosafety level 3 laboratories (BSL-3) are needed, limiting many countries from performing research and developing the vaccines independently.

### Recombinant Protein Vaccine

According to the statistics of WHO, among the COVID-19 vaccines being developed using different platforms, about 1/3rd of vaccines in clinical trials are recombinant protein vaccines (3). The expression systems used for COVID-19 recombinant protein vaccines in clinical and preclinical phase include nearly all currently available expression systems, such as yeast*, E. coli*, Expi293F cells, CHO cells, tobacco, and insect cells. The types of antigens expressed for the vaccines include RBD-monomer, RBDdimer, RBD-trimer, S1 protein, S protein and S-trimer. Vaccine adjuvants like Al(OH)3, AS01/AS03, Quil-A, monophosphoryl lipid A(MPL), Freund’s complete adjuvant, CpG, and Matrix M, Advax™, AgnHB have been tested (3, 40–45). Novavax’s NVX-Cov2373 leads ahead among protein subunit vaccines. It contains a prefusion S-trimer antigen with common PP substitutions and mutations at the S1–S2 polybasic cleavage site from RRAR to SRAG or QQAQ to render it protease resistant (46). Clover’s vaccine SCB-2019 is also a S-trimer vaccine. Different with NVX-Cov2373, the preparation of SCB-2019 relies on antigen fused in-frame to Trimer-Tag and subsequent self-trimerization of Trimer-Tag (47). While ZF2001, another leading protein vaccine, comprises a homogeneous RBD-dimers as a tandem-repeat single chain (40). Such antigen designs highly improved the immunogenicity and stability of subunit vaccines. Many developers and manufactures are focusing on developing recombinant protein vaccines because of the following advantages: (i) Specialized facilities like BSL-3 are not required and the expression of the recombinant protein can be easily scaled up. (ii) Adjuvants can be added to the formulation to bolster immunogenicity. (iii) The vaccines have good safety profiles since there are no live components; reactogenicity and side effects are mainly related to adjuvants.

### Live Attenuated Vaccine

Live attenuated vaccines (LAV) can be prepared and manufactured by means of “cold adaptation”, recombination, or reverse genetics. However, the time required for the processing of the virus is long. Currently, only one LAV has entered clinical trials, and two others are undergoing preclinical studies (48). COVI-VAC (NCT04619628), which is being developed by the Serum Institute of India and the American biotechnology company Codagenix, is in the advanced stages of development. COVI-VAC utilizes a computer algorithm to recode and de-optimize the SARS-CoV-2 virus specifically by introducing hundreds of mutations in the viral genome and removing the furin cleavage site (49). A similar technology has been used in the development of Respiratory syncytial virus (RSV), influenza, and dengue fever vaccines, and the safety of such an approach has been confirmed. Its advantages include: (i) The vaccine protein structure is identical to that of the live virus particles, and can induce immunity against total proteins from the whole virus. (ii) The risk of disease enhancement is low. However, LAV requires an extensive accessory testing regimen for establishing safety and efficacy. There are currently no clinical safety and effectiveness data for these kinds of COVID-19 vaccines.

## Efficacy, Immunogenicity, and Safety of Vaccines Produced From Different COVID-19 Vaccine Platforms

### Vaccine Efficacy

At present, most developers & manufacturers set the laboratory-confirmed COVID-19 as the primary endpoint for Phase 3 clinical trials, which is the most effective indicator to evaluate the protective efficacy of vaccines. At the end of 2020, protection data of 8 vaccines from 4 platforms have been published. The efficacy of these vaccines, referred to as VE henceforth, ranges from 50.38% to 95%. Remarkable VEs of 95% for Pfizer mRNA vaccine, and 94.1% for Moderna mRNA vaccine have been reported (50, 51). Adenovirus vectored vaccine Sputnik V, Ad26.COV2.S and Ad5-nCoV exhibited VE of 91.6%, 66% and 65.7%, respectively (52). Interestingly, AstraZeneca reported a 90% VE for its adenovirus vectored vaccine when a low dose prime, and standard dose boost regimen was employed, which went down to 62.1% for a standard dose prime and standard dose boost regimen of vaccination (53). The efficacy of Novavax recombinant subunit vaccine reported for the phase 3 clinical trial conducted in UK was 89.3% (54). while those for the inactivated vaccines of Sinopharm and Sinovac were 79.34% and 50.38%, respectively (55).

The following highlights emerge from the currently published VEs -

1) All reported VEs are higher than the 50% criterion required by WHO, FDA and EMA, indicating that the currently developed COVID-19 vaccines are efficacious against symptomatic COVID-19 in early stage (about 2-3 months) after vaccination (56–58). However, the long-term protective effect of the approved vaccines is currently unknown.
2) The VE reported for elderly population is relatively lower. This is concerning because the elderly are more susceptible to SARS-CoV-2 and show a higher death rate (59, 60). Most published data for current vaccines show relatively lower VE for the elderly (50, 53). Moderna mRNA vaccine showed a 95.6% VE for people younger than 65, but the VE dropped to 86.4% for people over 65. Pfizer mRNA vaccine showed a VE of 94.7% in the elderly population over 65, which is comparable to that observed in younger population (51).
3) The evidences for VE can be unreliable due to complicated demographic characteristics, such as population and geography. Exploratory subgroup analysis of BNT162b2 (Pfizer-BioNTech) showed a lower VE in Brazilian recipients (87.7%) than those in US (94.9%) and Argentinian recipients (97.2%) (51). The VE of CoronaVac (Sinovac) was reported as 91.25% in Turkey and 86% in UAE, respectively. While Brazil announced an overall 50.38% efficacy against COVID-19 for CoronaVac, and 78% against mild symptoms, which is far lower than that reported in Turkey (61). Above results indicate that the differences caused by population or geography do exist. AZD1222 (AstraZeneca) showed similar vaccine efficacy in SD/SD group in UK and Brazilian recipients, generalized from two diverse settings with different timings for the booster dose. The immunization interval between the two doses in the UK trial was mostly >12 weeks, while that in Brazil was less than 6 weeks. Interestingly, a significantly different efficacy of protection was exhibited by the first standard dose during immunization in the above two nations (71.2% in Brazil and 55% in UK). However, vaccine efficacy in UK recipients reached a comparable level with that in Brazil after the booster dose. Therefore, it is likely that a longer interval between the two doses of the vaccine might increase the immune protection (53).
4) VEs were affected by locally circulating variants. In the end of 2020, South Africa announced the emergency of a new variant B.1.351 (62, 63), and P.1 variant (64) was also found being prevalent in Brazil. Both variants contain mutations of E484K, K417T, and N501Y. E484K have been shown to reduce neutralization sensitivity of vaccine sera against SARS-CoV-2 (65, 66). NVX-CoV2373 developed by Novavax resulted in a point estimate of total VE of 89.3%, while 49.4% was reported in a Phase 2b clinical trial conducted in South Africa (67). Janssen’s vaccine Ad26.COV2.S resulted in 66% effective overall in preventing moderate to severe COVID-19, and reduced to 57% in South Africa (68). Recently, AstraZeneca-Oxford conducted a multicenter, double-blind, randomized, controlled trial to assess the safety and efficacy of ChAdOx1 nCoV-19 in South Africa, dedicating a VE of 21.9% overall and 10.4% against B.1.351 (31). Therefore, the circulating variants combined with geography factor greatly affect the calculated VE of vaccine candidates.
5) The dose and dosage influence the efficacy of the vaccine. The virus vectored vaccine is generally considered for application as a single-dose regimen to avoid compromising the immunogenicity of the second dose of the vaccine by the immune response against the viral vector elicited by the first dose. Although the AstraZeneca adenovirus vectored vaccine used a two-dose immunization regimen, the interim phase 3 clinical report showed that one standard dose provided similar protection as two standard doses regimen, with 55% (1 dose) and 60.3% (2 doses) VE in the UK recipients, and 71.2% (1 dose) and 64.2% (2 doses) VE in Brazilian recipients (53). Interestingly, cutting down half of the first dose of AZD1222 induced a protection of 90%, which was higher than the VE of 60.3% provided by two standard doses regimen in UK recipients. These statistics suggest that the adenovirus vaccine immunization schedule could be further optimized.

### Immunogenicity

#### Neutralizing Antibody

Neutralizing antibody (NtAb) titer is the most common correlate of protection against viral vaccines. The NtAb titer is highly correlated with protective effect and the durability of the protection. Results of previous studies on monoclonal antibodies and convalescent sera, as well as the tests conducted in animal models, have all confirmed the role of neutralizing antibodies in conferring protection against COVID-19 (69–71). Lilly’s neutralizing antibody bamlanivimab (LY-CoV555) has received the FDA emergency use authorization for the treatment of recently diagnosed COVID-19, and the data of phase 3 clinical trials have confirmed the potential of NtAbs in the prevention of COVID-19 (72, 73). Neutralizing antibody responses to SARS-CoV-2 in COVID-19 inpatients and convalescent patients were tested and the results showed that the NtAb titers were above 160 in more than 93% of the convalescent sera (74, 75). According to the results of clinical trials, the Geometric Mean Titer (GMT) of NtAb vary for different vaccine candidates. The recombinant protein vaccines developed by Clover and Novavax induced the highest neutralizing antibody GMTs of 3320 and 3906, respectively (76, 77). Comparatively, the GMTs of mRNA vaccines like those of Moderna and Pfizer stand at 654.3 and 361, respectively (50, 51, 78, 79). The neutralizing antibody GMT induced by various inactivated vaccines falls in the range of 50-300 (39, 80–82) (**Table 1**).

**Table 1:** Summarization of the clinical data for current advanced COVID-19 vaccine candidates.

Platforms	Vaccines (Developers)	Phase	Regimen	Efficacy (%)	Immunogenicity		Adverse Reactions (%)	
					NtAb	T cell:IFN-γ (SFUs/1×10^6^)	Local	Systemic
mRNA	mRNA -1273 (Moderna) (50, 78)	3	Day 0 + 28 100μg	94.1	654.3 (PRNT80)	0.1% (flowcytometry)	1^st^: 84.2 2^nd^: 88.9	1^st^: 54.9 2^nd^: 79.4
	BNT162b2 (BioNTech) (51, 79)	3	Day 0 + 28 30μg	95.0	361 (mNG-NT50)	1000	1^st^: ≥83 2^nd^: ≥78	1^st^: ≥47 2^nd^: ≥59
Adenovirus vectored	AZD1222 (AstraZeneca) (30, 53)	3	Day 0 + 28 5×10^10^vp	70.4 (Total) 90 (LD/SD) 60.3 (SD/SD)	161-193 (MNT_80_)	797-1187	88	86
	Ad5-nCoV (CanSino) (32, 83)	3	Day 0 5×10^10^vp	65.7	18.3 (MNT_50_)	100	≥56	≥44
	Sputnik V (Gamaleya) (33)	3	Day 0 + 21 1×10^11^vp	91.6	49.25 (MNT_50_)	1.3% (flowcytometry)	≥40	≥95
	Ad26.COV2.S (Janssen Pharm) (34)	3	Day 0 + 56 5×10^10^vp	66	1^st^: 277-321 2^nd^: 827 (MNT_50_)	0.08%-0.09%	64	65
Inactivated	BBIBP-CorV (Beijing, Sinopharm) (80)	3	Day 0 + 21 4μg	79.34	282.7 (MNT_50_)	—	≥12 and ≤18	≥4 and ≤18
	CoronaVac (Sinovac) (82)	3	Day 0 + 14 Day 0 + 28 3μg	91.25 (Turkey) 86 (UAE) 50.38 (Brazil)	65.4 (MNT_50_)	55	16.7-23.3	12.5-15.8
	Inactivated (Wuhan, Sinopharm) (81)	3	Day 0 + 21 5μg	72.51	247 (PRNT_50_)	—	15.5	4.8
	BBV152 (Bharat Biotech) (39)	3	Day 0 + 14 6μg	—	66.4(MNA_50_) ≈120(PRNT_50_)	55	5	14
Recombinant subunit	NVX-CoV2373 (Novavax) (76)	3	Day 0 + 21 5μg	89.3	3906 (MNT_99_)	≈0%-1.5% (flowcytometry)	1^st^: 69.2 2^nd^: 91.7	1^st^: 46.1 2^nd^: 65.4
	ZF2001 (Zhifei Longcom) (84)	3	Day 0 + 28+56 25μg	—	102.5 (MNT_50_)	≈8	≥9 and ≤48	≥8 and ≤48
	SCB-2019 (Clover) (77)	2/3	Day 0 + 21 9μg	—	1810-3320 (MNT_50_)	≈0.1% (flowcytometry)	1^st^: 50 2^nd^: 68.8	1^st^: 25 2^nd^: 56.3
DNA	INO-4800 (Inovio) (27)	2/3	Day 0 + 28 1mg	—	102.3 (PRNT_50_)	46	10 (vaccine related)	0 (vaccine related)

1. NtAb, Neutralizing Antibody; SFUs, Spot Forming Units; MNT, Micro-neutralization Test; PRNT, Plaque Reduction Neutralization Test.

2. All data were collected from the current released reports for each vaccine. The measurement methods and detection virus strains are different for each vaccine candidate, and the results are incomparable. The table is for reference only.

Differences in the type of viral strains and methods used for NtAb measurement, hamper the direct comparison of the results obtained by different labs and introduce bias in the real immunogenicity data of COVID-19 vaccine candidates. Comparing vaccine-induced NtAb with convalescent sera can eliminate the differences caused by different materials and methods used to a certain extent. Accordingly, we summarized the neutralizing antibody data for vaccinated and convalescent sera from clinical trial reports (**Table 1**) and the ratios of NtAb GMTs of vaccinated sera to convalescent sera were calculated. The descending order of the vaccines based on the resultant ratios starting from the highest to the lowest is as follows - mRNA-1273, NVX-CoV2373, BNT162b2, SCB-2019+AS03, AZD1222, ZF2001, Ad26.COV2.S, Sputnik V, BBV152, BBIBP-CorV, and CoranaVac. The numbers for convalescent sera for the inactivated vaccine from Sinopharm (Wuhan), Ad5-nCoV from Cansino and INO-4800 from Inovio were unavailable during the time of the calculation of the ratios. A rough analysis by our group shows that the efficacy of current vaccine candidates is related to the levels of GMT of neutralizing antibody to some degree, but the lack or inconsistency of convalescent sera control might make the correlation unreliable.

The neutralizing antibody level induced by different vaccines originating from the same platform vary. The impediments in evoking strong immune responses by vaccines can be overcome by optimizing antigens, adjuvants, immunization schemes, manufacturing processes, etc.

1) The levels of neutralizing antibodies induced by the three adenovirus vectored vaccines are quite different due to the different types of vectors and immunization procedures applied in clinical trials (1 dose for Ad5; 2 doses for ChAdOx1; 1-2 doses for Ad26; 1 dose each for Ad5 and Ad26). Data from the published reports of the clinical trials shows that the neutralizing antibody GMT varies from 18·3 to 827 for these vaccines (30, 32, 33, 85).
2) Moderna’s mRNA technology has been previously applied for the development of vaccines against influenza and Zika viruses (86, 87). Those vaccines showed poor immunogenicity at the early stage, owing to sub-optimal antigen design. The optimized candidate later showed better immunogenicity. Thus, the first generation of COVID-19 mRNA vaccine could be further improved by optimization of the primary sequence of antigens and trying out different immunization regimens.
3) The immunogenicity of recombinant COVID-19 protein vaccine can be improved by novel adjuvant. Novavax utilizes a new adjuvant Matrix M. The NtAb GMT of low dose group for this vaccine formulation was 3906 (76). Clover, in collaboration with GSK developed an AS03 adjuvanted subunit vaccine, and the NtAb level of high-dose group reached 3900 for this vaccine (77). Both vaccines adjuvanted by novel adjuvants seem to elicit higher NtAbs than ZF2001, which adjuvanted by a classical but much safer Alum adjuvant.

#### Cellular Immunity

Cellular immunity is necessary for generation of neutralizing antibodies and plays an important role in controlling SARS-CoV-2 infection (71). The Th1 immune response is set as one of the key points for vaccine evaluation by WHO Candidate Vaccine Prioritization Working Group (13). We compared the capacity of several vaccines to induce IFN-γ-secreting T-cell response, and the results showed that adenovirus vectored vaccines and mRNA vaccines induced relatively strong cellular immune responses (SFU:1187-100) (**Table 1**), higher than those of other types of vaccines. Comparatively, the two inactivated vaccines reported a T-cell response data that were relatively weaker (SFU:55) (**Table 1**). The recombinant protein vaccines vary in activating T-cell responses and they are dependent on the different adjuvants used (**Table 1**). It is important to further verify the correlationship between activation of T-cell response and protective effect of COVID-19 vaccines.

### Safety

The safety profiles of different types of COVID-19 vaccines are quite different. The incidences of local and systemic adverse reactions (AR) are relatively higher in mRNA vaccines and adenovirus vaccines (Local: 88.9%-40%, Systemic: 86%-44%). The side-effects were lowest in inactivated vaccines (Local: 23.3%-5%, Systemic: 18%-4%). However, the AR rate of recombinant protein vaccines varies greatly as different adjuvants are used (Local: 91.7%-9%; Systemic: 65.4-8%). Safety and the level of neutralizing antibodies induced are inversely related, and the AR rate noted in the elderly is lower than that of adults.

Novel adjuvants and delivery materials used in mRNA vaccines and recombinant vaccines seem to increase side effects, especially after the second dose, which needs to be investigated further for improving the vaccine.

1) ARs noted for the Moderna and Pfizer-BioNTech mRNA vaccines increased after the second dose. The local and systemic ARs in the median dose groups of Moderna were 88.9%(Local), 79.4%(Systemic); and those for Pfizer vaccines were >79%(Local), and >59%(Systemic) (50, 51, 88). Severe allergic reactions have been reported after the administration of the two vaccines (89, 90). This raises a safety concern in population and may impact vaccination compliance. Coated liposomes are considered as the main cause of ARs, and how to improve the packaging and delivery system is crucial for enhancing the safety profile of mRNA vaccines (91). In addition, vaccine design can also affect vaccine safety. Pfizer-BioNTech designed two mRNA vaccines for clinical trials, namely BNT162b2 and BNT162b1. BNT162b1 encodes the trimerized receptor binding domain, while BNT162b2 encodes full length spike, modified by two proline mutations to lock it in the prefusion conformation (79). Phase 1 clinical data showed that the two vaccines induced similar dose-dependent neutralizing antibody titers, and the titer was equivalent to or higher than that of convalescent serum. However, the incidence and severity of systemic reactions caused by BNT162b2, especially in the elderly, were lower (79).
2) ARs of Novavax and Clover vaccine are significantly increased after the second dose. The AR rate of Novavax recombinant vaccine with Matrix M adjuvant (69.2% local; 46.1% systemic) was comparable with that of the Clover vaccine with GSK AS03 adjuvant (68.8% local and 56.3% systemic). The ARs of those two vaccines aforementioned are higher than that of Zhifei vaccine adjuvanted with alum (total ARs <48%) (76, 77, 84).

Antibody-dependent enhancement (ADE) and vaccine-associated disease enhancement (VADE) are two major public concerns in vaccine safety evaluation. ADE is mainly mediated by antibody Fc receptor-associated internalization of a virus. VADE is a disease situation associated vaccine, mainly attributed to antibody-dependent and type 2 T helper cell-dependent mechanisms. For safety consideration, a vaccine should be highly effective in triggering high level of neutralizing antibody and Th1 type cellular responses *in vivo*, and ADE as well as VADE should be monitored in clinical and preclinical studies (92). Fortunately, there is still no report revealing an ADE phenomenon in the development process of current candidate COVID-19 vaccines. Further studies should be conducted to clarify the potential risk caused by ADE and VADE during COVID-19 vaccines application.

## Criterion and Reference Standards Are Crucial in COVID-19 Vaccine Development and Evaluation

Defining successful vaccines as well as identifying and applying them widely are crucial for controlling the pandemic. A well-defined effective vaccine identified from the “first wave” will set a benchmark for the development of second-generation vaccines. Criterion for evaluation and standards for immune response measurement are two important considerations essential for efficient, speedy, and reliable evaluation of a large number of candidate vaccines against COVID-19.

### Criterion for COVID-19 Vaccine Evaluation

In order to ensure the effectiveness and safety of COVID-19 vaccines, WHO, FDA, EUA, and NMPA have issued guidance for industry at the early stage (56–58, 93), providing key considerations for vaccine development, quality control and clinical evaluation. Guidance points out that to ensure that a widely deployed COVID-19 vaccine is effective, the primary efficacy endpoint point estimate for a placebo-controlled efficacy trial should be at least 50%, and the statistical success criterion should be that the lower bound of the appropriately alpha-adjusted confidence interval around the primary efficacy endpoint point estimate is >30%. An inappropriate (relatively lower) criteria set for the first generation of COVID-19 vaccine can further influence the development of next generation vaccines.

The criterion for defining COVID-19 cases and the methods used for diagnosis have a profound impact on the final calculated vaccine efficacy (94, 95). At present, developers mainly use symptoms, nucleic acid testing, and serological testing for case determination, though there are no universal standards for case collection and inclusion. Under the circumstance that multi-countries and multi-enterprises are involved in COVID-19 vaccine research and development, the establishment of relatively consistent diagnostic and case collection criteria for clinical research can help obtain reliable and comparable efficacy data for vaccines.

### Standards for COVID-19 Vaccine Evaluation and Comparison

Although we comprehensively analyzed multiple platforms of COVID-19 vaccines based on the clinical research data published by various developers, the analyzed results could be unreliable and incomparable because of instances where no standards that could serve as reference points were incorporated in the studies and where different methods were used for estimating immunogenicity. This is a major obstacle in evaluating and prioritizing current COVID-19 vaccine candidates from the available data. The establishment and incorporation of reference standards for evaluating antibodies and antigens needs to be implemented.

Recently, a variety of molecular and serological assays have been developed for the detection of SARS-CoV-2 infection, the measurement of antibody response to SARS-CoV-2 infection and the tiers of antibodies induced by COVID-19 vaccines. The WHO collaboration center for biological standardization and the National Institute for Biological Standards and Control (NIBSC) recently organized and completed the collaborative calibration of mRNA and antibody standards (96–98), and endorsed a proposal to develop a standard for SARS-CoV-2 antigens to support the development, assessment and comparability of antigen-based rapid diagnostic tests (99).

1) The First WHO International Standard for SARS-CoV-2 RNA for nucleic acid amplification techniques (NAT) based assays was established by the Committee with assigned unitage of 7.40 log10 IU/ampoule. The NAT-based assay is considered the gold standard for accurate diagnosis of infection (98). The establishment of nucleic acid standard is of great significance not only for the facilitation of accurate diagnosis but also for the comparability of clinical trial data.
2) The First WHO International Standard for anti-SARS-CoV-2 immunoglobulin was established with an assigned unitage of 250 IU/ampoule (neutralizing antibody activity). And, the First WHO International Reference Panel of anti-SARS-CoV-2 immunoglobulin was also established with no assigned units. The availability of an International Standard for antibodies to SARS-CoV-2 would facilitate the standardization of SARS-CoV-2 serological methods and allow for comparison and harmonization of datasets across laboratories. This will help accurately determine whether the antibody levels that are needed for effective protection are elicited by vaccines. Previously, China’s NIFDC had established the first COVID-19 national standard for neutralizing antibody using convalescent serum, which can be used for vaccine evaluation. In the future, secondary standards may be established by other countries/regions. It is recommended that the National Regulatory Authorities (NRAs) and companies in vaccine production countries harmonize their own secondary standards or in-house standards to the international standards established by the WHO to facilitate parallel comparison of vaccine induced antibodies in different countries/regions.

## Sequential Immunization Strategy and Next Generation Vaccine Development

Since there is variation in (i) the protection conferred by various vaccines and (ii) the safety profile of vaccines developed for emergency use, alternative strategies to mitigate the shortcomings of the vaccines need to be investigated. Adoption of a sequential immunization strategy may make up for the deficiencies of existing vaccines to a certain extent. More recently, another important issue regarding emergence of mutant strains of SARS-CoV2 that have lowered the efficacy of the vaccines has cropped up. A safer and more effective next-generation COVID-19 vaccine that can effectively respond to virus mutants should be developed to counter the threat posed by viral variants.

### Sequential Immunization Strategy

An in-depth analysis of the clinical data released for the first-generation of COVID-19 vaccines unveils the following key issues: (1) The immune responses and protective efficacies in the elderly recipients are low, so are the adverse reaction rates. (2) mRNA and recombinant protein vaccines developed with new adjuvants produce high levels of neutralizing antibodies, but the adverse reactions are also high, especially after the second dose. (3) The inactivated vaccine has the lowest adverse reaction rate, but the overall immune response is not strong. (4) The T cell response of most adenovirus vaccines is relatively good, but the level of neutralizing antibodies is not high. How to strike a balance between the immunogenicity, effectiveness and safety of vaccines is a key issue that should be considered in the development of first-generation vaccines for emergency use.

The sequential vaccine immunization strategy may be an effective measure to solve the immunogenicity and safety problems of emergency vaccines and improve the duration of protection. This has been observed in studies on HIV vaccines. Development of an effective HIV vaccine is considered to be extremely difficult. In clinical research on HIV vaccines, there has never been a single vaccine that could provide effective protection. For the first time, the RV144 vaccine has passed the initial vaccination efficacy of poxvirus, and the sequential immunization strategy enhanced by the recombinant subunit vaccine has achieved 31.2% protection efficiency (100). This heterogeneous prime-boost strategy, also known as sequential immunization strategy, has gradually attracted attention of researchers. A large number of animal experiments have confirmed that sequential immunization with different types of vaccines can increase the intensity and breadth of the immune response (101).

The outbreak of the COVID-19 epidemic has promoted the rapid development of the vaccine industry. In just one year, more than one hundred COVID-19 vaccines from various platforms have entered clinical trials, which has laid the foundation for the research and application of sequential immunization strategies. The COVID-19 vaccines of different platforms have different immunological characteristics. The use of heterologous vaccines for sequential immunization can theoretically avoid boosting immunization of non-target proteins and enhance the strength of the immune response against the target antigen. Among the various vaccines currently being researched and marketed, some vaccines are mainly used to induce T cell responses, and some are mainly used to induce antibody responses. By using vaccines with different immune characteristics for sequential immunization, it is possible to obtain higher levels of antibody response and T cell response. In addition, sequential immunization may theoretically reduce the risk of ARs caused by a second dose of a single vaccine. In particular, reports have shown that the incidence of ARs increase when the second dose of mRNA vaccine is administered (78, 79). However, it should be noted that the US CDC has clearly pointed out that the safety and effectiveness of mixed vaccination of different types of vaccines have not been verified, and mixed vaccination is currently not permitted, except under special circumstances. Although availability of vaccines from different platforms makes a compelling case for sequential immunization strategies, further studies are required to ascertain the benefits of such a strategy for COVID-19.

### Next Generation Vaccine Development

The mRNA vaccine has more than 90% protection efficacy against COVID-19 (50, 51). The underly mechanism involves introducing the gene of the spike protein inside the host cell, indicating that the immune response against the spike protein can provide effective protection. However, the mutation-prone nature of Spike protein puts a question mark on the duration of vaccine protection and the effectiveness of the vaccine in the future. Since the start of the COVID-19 epidemic, four variants have been found, namely D614G (102) that appeared in late January-February 2020, cluster 5 found in Danish mink from June to August, 2020, B.1.1.7 “N501Y.V1”reported on December 14, 2020, in the United Kingdom (103), and B.1.351 “501Y.V2” (104), that appeared on December 18, 2020, in South Africa. Except for cluster 5, rest of the three mutant strains caused a significant increase in the infectivity of the virus. Neutralization studies showed that the convalescent serum’s ability to neutralize B.1.351 was compromised, while the B.1.1.7 mutant strain was still sensitive to neutralizing antibodies induced by ancestral Spike vaccines (104–107). There is still concern whether the first-generation vaccines can provide sufficient protection against the mutant strains. Sera from recipients vaccinated 3 weeks following the first dose of Pfizer-BioNTech’s BNT162b2 vaccine showed reduced neutralizing titers against the full set of Spike mutations present in the B.1.1.7 variant (108). Neutralizing ability of the mRNA-1273 (Moderna) vaccinated sera against variants were also tested using pseudovirus-based neutralization assay. The results showed that the neutralizing ability of mRNA-1273 vaccinated serum had no significant changes for B.1.1.7, but dropped for B.1.351 (109). The recombinant subunit vaccine NVX-CoV2373 showed protective efficacy of 89.3% in phase 3 clinical trial conducted in the United Kingdom and about 50% of infected recipients turned out to be B.1.1.7 variant positive. Further analysis of UK clinical data showed that NVX-CoV2373 has an efficacy of 95.6% against the ancestral strain and 85.6% against the UK variant. However, the efficacy in South African recipients dropped to 60% because 90% of those recipients turned out to be B.1.351 variant positive (54). At present, many other companies are testing the neutralizing ability of their vaccine serum against variant strains, which should also become a very important consideration in vaccine evaluation.

Developers and manufacturers should focus their efforts on viral mutations and propose next generation vaccine development strategies to deal with mutants. The next generation COVID-19 vaccine should be recoded and prepared according to circulating variants sequences. Alternatively, immunogenetic conserved virus proteins, linear B cell epitopes and MHC I/II restricted T cell epitopes screened by bioinformatics approach (110–112) might provide novel vaccine ideas for next generation vaccine design to combat continuously virus mutation. The production processes of vaccines for each platform differ. Recombinant nucleic acid vaccines and recombinant virus vectored vaccines enter cells to express antigens to stimulate the immune system. These types of vaccines can be produced by introducing new sequences of antigens into the genome and utilizing the existing production facilities for manufacturing. In principle, as long as the new sequence information is available, the vaccine can be redesigned and synthesized. Because of the speed offered in design and synthesis as well as the good immunogenicity of mRNA vaccine, it stands out as the platform of choice for the urgent development of COVID-19 vaccines. But, the uniqueness of the technology limits the research and development by many companies/institutes (23, 24). Compared to mRNA vaccine, the production process for recombinant subunit vaccine is relatively complicated, requiring transcription in *in vitro* to generate immunogenic proteins. Adjustment of the manufacturing processes for producing mutant proteins is not as straight-forward and introduction of mutations could change the immunogenicity of the protein. Inactivated vaccines require the isolation of the new mutant strains in BSL-3 laboratories before they can be amplified and inactivated to develop vaccines for emerging novel variants. High-level biosafety production facilities and strain resources are the main constraints (113, 114).

## Conclusions

The COVID-19 pandemic is an event that has changed human lifestyle and history. The development and application of COVID-19 vaccines have also greatly changed the understanding of vaccines, as well as led to revolutionary changes in the process of vaccine development. The first generation of COVID-19 vaccines developed for emergency use have shown promise in controlling the pandemic. Several deficiencies of the current lot of vaccines are starting to emerge; in particular, the decreased efficacy in elderly, higher incidences of adverse reactions noted for some vaccines and reduced protection against mutant strains are of concern. Sequential immunization strategy that has been studied for many infectious disease vaccines might help mitigate many of the shortcomings of the current lot of COVID-19 vaccines. A key focus for the development of next generation of vaccines would be enhanced protection against newly emerging mutants. Standardization of measurements is essential for comparison of efficacies of different vaccines. Regulatory authorities have already established guidelines for the development and evaluation of COVID-19 vaccines. They have also introduced standards that could serve as references for evaluating the efficacy of vaccines. When implemented in clinical studies, these could facilitate the comparison of the results of vaccines and aid in the prioritization of vaccine candidates for mass vaccination campaigns.

## Author Contributions

ZL, MX, and JW conceived the framework and main text of this review article. QH and ZL wrote the draft. QM, LB, and FG reviewed the manuscript. JZ searched the literature. All authors contributed to the article and approved the submitted version.

## Funding

This work was supported by the Major Special Projects Funding Program (No. 2020YFC0860500) of the Ministry of Science and Technology of the People’s Republic of China.

## Conflict of Interest

The authors declare that the research was conducted in the absence of any commercial or financial relationships that could be construed as a potential conflict of interest.

## References

[1] World Health Organization. WHO Coronavirus Disease (COVID-19) Dashboard (2021). Available at: https://covid19.who.int/ (Accessed February 15, 2021).

[2] Ball P. The lightning-fast quest for COVID vaccines — and what it means for other diseases. Nature (2020). 10.1038/d41586-020-03626-1 33340018

[3] World Health Organization. The COVID-19 candidate vaccine landscape (2021). Available at: https://www.who.int/publications/m/item/draft-landscape-of-covid-19-candidate-vaccines (Accessed February 15, 2021).

[4] Food and Drug Administration. COVID-19 Vaccines Authorized for Emergency Use- Pfizer-BioNTech COVID-19 Vaccine. Available at: https://www.fda.gov/emergency-preparedness-and-response/coronavirus-disease-2019-covid-19/pfizer-biontech-covid-19-vaccine (Accessed December 11, 2020).

[5] Food and Drug Administration. COVID-19 Vaccines Authorized for Emergency Use-Moderna COVID-19 Vaccine (2020). Available at: https://www.fda.gov/emergency-preparedness-and-response/coronavirus-disease-2019-covid-19/moderna-covid-19-vaccine (Accessed December 18, 2020).

[6] Medicines and Healthcare products Regulatory Agency. UK medicines regulator gives approval for first UK COVID-19 vaccine (2020). Available at: https://www.gov.uk/government/news/uk-medicines-regulator-gives-approval-for-first-uk-covid-19-vaccine (Accessed December 2, 2020).

[7] Medicines and Healthcare products Regulatory Agency. Oxford University/AstraZeneca COVID-19 vaccine approved (2020). Available at: https://www.gov.uk/government/news/oxford-universityastrazeneca-covid-19-vaccine-approved (Accessed December 30, 2020).

[8] European Medicines Agency. EMA recommends first COVID-19 vaccine for authorisation in the EU (2020). Available at: https://www.ema.europa.eu/en/news/ema-recommends-first-covid-19-vaccine-authorisation-eu (Accessed December 21, 2020).

[9] Russian Direct Investment Fund. Sputnik V authorized in 26 countries (2021). Available at: https://sputnikvaccine.com/newsroom/pressreleases/sputnik-v-authorized-in-26-countries/ (Accessed February 12, 2021).

[10] European Medicines Agency. EMA recommends COVID-19 Vaccine Moderna for authorisation in the EU (2021). Available at: https://www.ema.europa.eu/en/news/ema-recommends-covid-19-vaccine-moderna-authorisation-eu (Accessed January 6, 2021).

[11] Coronavirus Today. COVID-19 VACCINES (2021). Available at: https://www.coronavirustoday.com/covid-19-vaccines (Accessed February 16, 2021).

[12] World Health Organization. WHO issues its first emergency use validation for a COVID-19 vaccine and emphasizes need for equitable global access(WHO) (2020). Available at: https://www.who.int/news/item/31-12-2020-who-issues-its-first-emergency-use-validation-for-a-covid-19-vaccine-and-emphasizes-need-for-equitable-global-access (Accessed December 31, 2020).

[13] World Health Organization. Criteria for COVID-19 vaccine prioritization (2020). Available at: https://www.who.int/publications/m/item/criteria-for-covid-19-vaccine-prioritization (Accessed May 27 2020).

[14] Krause P Fleming TR Longini I Henao-Restrepo AM Peto R Dean NE. COVID-19 vaccine trials should seek worthwhile efficacy. Lancet (2020) 396:741–3. 10.1016/s0140-6736(20)31821-3 PMC7832749 32861315

[15] Amanat F Krammer F. SARS-CoV-2 Vaccines: Status Report. Immunity (2020) 52:583–9. 10.1016/j.immuni.2020.03.007 PMC7136867 32259480

[16] Lurie N Saville M Hatchett R Halton J. Developing Covid-19 Vaccines at Pandemic Speed. N Engl J Med (2020) 382:1969–73. 10.1056/NEJMp2005630 32227757

[17] Song Z Xu Y Bao L Zhang L Yu P Qu Y. Viruses From SARS to MERS, Thrusting Coronaviruses into the Spotlight. Viruses (2019) 11:59. 10.3390/v11010059 PMC6357155 30646565

[18] Cai Y Zhang J Xiao T Peng H Sterling SM Walsh RM Jr. Distinct conformational states of SARS-CoV-2 spike protein. Science (2020) 369:1586–92. 10.1126/science.abd4251 PMC7464562 32694201

[19] Dai L Gao GF. Viral targets for vaccines against COVID-19. Nat Rev Immunol (2020) 21:73–82. 10.1038/s41577-020-00480-0 33340022 PMC7747004

[20] Huo J Zhao Y Ren J Zhou D Duyvesteyn HME Ginn HM. Neutralization of SARS-CoV-2 by Destruction of the Prefusion Spike. Cell Host Microbe (2020) 28:445–54.e446. 10.1016/j.chom.2020.06.010 32585135 PMC7303615

[21] Zhou D Duyvesteyn HME Chen CP Huang CG Chen TH Shih SR. Structural basis for the neutralization of SARS-CoV-2 by an antibody from a convalescent patient. Nat Struct Mol Biol (2020) 27:950–8. 10.1038/s41594-020-0480-y 32737466

[22] Medicines and Healthcare products Regulatory Agency. Moderna vaccine becomes third COVID-19 vaccine approved by UK regulator (2021). Available at: https://www.gov.uk/government/news/moderna-vaccine-becomes-third-covid-19-vaccine-approved-by-uk-regulator (Accessed January 8, 2021).

[23] Vogel AB Kanevsky I Che Y Swanson KA Muik A Vormehr M. A prefusion SARS-CoV-2 spike RNA vaccine is highly immunogenic and prevents lung infection in non-human primates. bioRxiv (2020). 10.1101/2020.09.08.280818

[24] Corbett KS Edwards D Leist SR Abiona OM Boyoglu-Barnum S Gillespie RA. SARS-CoV-2 mRNA Vaccine Development Enabled by Prototype Pathogen Preparedness. bioRxiv (2020). 10.1101/2020.06.11.145920 PMC7581537 32756549

[25] Maruggi G Zhang C Li J Ulmer JB Yu D. mRNA as a Transformative Technology for Vaccine Development to Control Infectious Diseases. Mol Ther (2019) 27:757–72. 10.1016/j.ymthe.2019.01.020 PMC6453507 30803823

[26] Sharma O Sultan AA Ding H Triggle CR. A Review of the Progress and Challenges of Developing a Vaccine for COVID-19. Front Immunol (2020) 11:585354. 10.3389/fimmu.2020.585354 33163000 PMC7591699

[27] Tebas P Yang S Boyer JD Reuschel EL Patel A Christensen-Quick A. Safety and immunogenicity of INO-4800 DNA vaccine against SARS-CoV-2: A preliminary report of an open-label, Phase 1 clinical trial. EClinicalMedicine (2020) 31:100689. 10.1016/j.eclinm.2020.100689 33392485 PMC7759123

[28] Zhu FC Hou LH Li JX Wu SP Liu P Zhang GR. Safety and immunogenicity of a novel recombinant adenovirus type-5 vector-based Ebola vaccine in healthy adults in China: preliminary report of a randomised, double-blind, placebo-controlled, phase 1 trial. Lancet (2015) 385:2272–9. 10.1016/s0140-6736(15)60553-0 25817373

[29] First Ebola vaccine approved. Nat Biotechnol (2020) 38:6. 10.1038/s41587-019-0385-7 31919440

[30] Folegatti PM Ewer KJ Aley PK Angus B Becker S Belij-Rammerstorfer S. Safety and immunogenicity of the ChAdOx1 nCoV-19 vaccine against SARS-CoV-2: a preliminary report of a phase 1/2, single-blind, randomised controlled trial. Lancet (2020) 396:467–78. 10.1016/s0140-6736(20)31604-4 PMC7445431 32702298

[31] Madhi SA Baillie V Cutland CL Voysey M Koen AL Fairlie L. Efficacy of the ChAdOx1 nCoV-19 Covid-19 Vaccine against the B.1.351 Variant. N Engl J Med (2021). 10.1056/NEJMoa2102214 PMC7993410 33725432

[32] Zhu F-C Guan X-H Li Y-H Huang J-Y Jiang T Hou L-H. Immunogenicity and safety of a recombinant adenovirus type-5-vectored COVID-19 vaccine in healthy adults aged 18 years or older: a randomised, double-blind, placebo-controlled, phase 2 trial. Lancet (2020) 396:479–88. 10.1016/s0140-6736(20)31605-6 PMC7836858 32702299

[33] Logunov DY Dolzhikova IV Zubkova OV Tukhvatulin AI Shcheblyakov DV Dzharullaeva AS. Safety and immunogenicity of an rAd26 and rAd5 vector-based heterologous prime-boost COVID-19 vaccine in two formulations: two open, non-randomised phase 1/2 studies from Russia. Lancet (2020) 396:887–97. 10.1016/s0140-6736(20)31866-3 PMC7471804 32896291

[34] Sadoff J Le Gars M Shukarev G Heerwegh D Truyers C de Groot AM. Interim Results of a Phase 1-2a Trial of Ad26.COV2.S Covid-19 Vaccine. N Engl J Med (2021). 10.1056/NEJMoa2034201 PMC7821985 33440088

[35] Bos R Rutten L van der Lubbe JEM Bakkers MJG Hardenberg G Wegmann F. Ad26 vector-based COVID-19 vaccine encoding a prefusion-stabilized SARS-CoV-2 Spike immunogen induces potent humoral and cellular immune responses. NPJ Vaccines (2020) 5:91. 10.1038/s41541-020-00243-x 33083026 PMC7522255

[36] Zhang C Zhou D. Adenoviral vector-based strategies against infectious disease and cancer. Hum Vaccin Immunother (2016) 12:2064–74. 10.1080/21645515.2016.1165908 PMC4994731 27105067

[37] Geisbert TW Bailey M Hensley L Asiedu C Geisbert J Stanley D. Recombinant adenovirus serotype 26 (Ad26) and Ad35 vaccine vectors bypass immunity to Ad5 and protect nonhuman primates against ebolavirus challenge. J Virol (2011) 85:4222–33. 10.1128/jvi.02407-10 PMC3126236 21325402

[38] Merck Discontinues Development of SARS-CoV-2/COVID-19 Vaccine Candidates (2021). Available at: https://www.merck.com/news/merck-discontinues-development-of-sars-cov-2-covid-19-vaccine-candidates-continues-development-of-two-investigational-therapeutic-candidates/ (Accessed January 25, 2021).

[39] Ella R Mohan K Jogdand H Prasad S Reddy S Sarangi VK. Safety and Immunogenicity Trial of an Inactivated SARS-CoV-2 Vaccine BBV152. medRxiv (2020). 10.1101/2020.12.11.20210419

[40] Dai L Zheng T Xu K Han Y Xu L Huang E. A Universal Design of Betacoronavirus Vaccines against COVID-19, MERS, and SARS. Cell (2020) 182:722–33.e711. 10.1016/j.cell.2020.06.035 32645327 PMC7321023

[41] Liu Z Xu W Xia S Gu C Wang X Wang Q. RBD-Fc-based COVID-19 vaccine candidate induces highly potent SARS-CoV-2 neutralizing antibody response. Signal Transduct Target Ther (2020) 5:282. 10.1038/s41392-020-00402-5 33247109 PMC7691975

[42] Powell AE Zhang K Sanyal M Tang S Weidenbacher PA Li S. A single immunization with spike-functionalized ferritin vaccines elicits neutralizing antibody responses against SARS-CoV-2 in mice. bioRxiv (2020). 10.1101/2020.08.28.272518 PMC7805605 33527087

[43] Su Q Zou Y Yi Y Shen L Ye C Zhang Y. Recombinant SARS-CoV-2 RBD molecule with a T helper epitope as a built in adjuvant induces strong neutralization antibody response. bioRxiv (2020). 10.1101/2020.08.21.262188 PMC7816590 33516600

[44] Yang J Wang W Chen Z Lu S Yang F Bi Z. A vaccine targeting the RBD of the S protein of SARS-CoV-2 induces protective immunity. Nature (2020) 586:572–7. 10.1038/s41586-020-2599-8 32726802

[45] Powell AE Zhang K Sanyal M Tang S Weidenbacher PA Li S. A Single Immunization with Spike-Functionalized Ferritin Vaccines Elicits Neutralizing Antibody Responses against SARS-CoV-2 in Mice. ACS Cent Sci (2021) 7:183–99. 10.1021/acscentsci.0c01405 PMC7805605 33527087

[46] Tian JH Patel N Haupt R Zhou H Weston S Hammond H. SARS-CoV-2 spike glycoprotein vaccine candidate NVX-CoV2373 immunogenicity in baboons and protection in mice. Nat Commun (2021) 12:372. 10.1038/s41467-020-20653-8 33446655 PMC7809486

[47] Liang JG Su D Song TZ Zeng Y Huang W Wu J. S-Trimer, a COVID-19 subunit vaccine candidate, induces protective immunity in nonhuman primates. Nat Commun (2021) 12:1346. 10.1038/s41467-021-21634-1 33649323 PMC7921634

[48] Seo SH Jang Y. Cold-Adapted Live Attenuated SARS-Cov-2 Vaccine Completely Protects Human ACE2 Transgenic Mice from SARS-Cov-2 Infection. Vaccines (Basel) (2020) 8:584. 10.3390/vaccines8040584 PMC7712048 33022950

[49] Arthur R. Needle-free COVIC-19 vaccine COVI-VAC enters Phase 1 trials (2020). Available at: https://www.biopharma-reporter.com/Article/2021/01/11/Needle-free-COVID-19-vaccine-COVI-VAC-enters-Phase-1-trials (Accessed January 11, 2021).

[50] Baden LR El Sahly HM Essink B Kotloff K Frey S Novak R. Efficacy and Safety of the mRNA-1273 SARS-CoV-2 Vaccine. N Engl J Med (2020) 384:403–16. 10.1056/NEJMoa2035389 PMC7787219 33378609

[51] Polack FP Thomas SJ Kitchin N Absalon J Gurtman A Lockhart S. Safety and Efficacy of the BNT162b2 mRNA Covid-19 Vaccine. N Engl J Med (2020) 383:2603–15. 10.1056/NEJMoa2034577 PMC7745181 33301246

[52] Russian Direct Investment Fund. Second interim analysis of clinical trial data showed a 91.4% efficacy for the Sputnik V vaccine on day 28 after the first dose; vaccine efficacy is over 95% 42 days after the first dose (2020). Available at: https://sputnikvaccine.com/newsroom/pressreleases/second-interim-analysis-of-clinical-trial-data-showed-a-91-4-efficacy-for-the-sputnik-v-vaccine-on-d/ (Accessed 2020).

[53] Voysey M Clemens SAC Madhi SA Weckx LY Folegatti PM Aley PK. Safety and efficacy of the ChAdOx1 nCoV-19 vaccine (AZD1222) against SARS-CoV-2: an interim analysis of four randomised controlled trials in Brazil, South Africa, and the UK. Lancet (2020) 397:99–111. 10.1016/s0140-6736(20)32661-1 33306989 PMC7723445

[54] Novavax. Novavax COVID-19 Vaccine Demonstrates 89.3% Efficacy in UK Phase 3 Trial (2021). Available at: https://ir.novavax.com/news-releases/news-release-details/novavax-covid-19-vaccine-demonstrates-893-efficacy-uk-phase-3 (Accessed January 28, 2021).

[55] Sinopharm. China grants conditional market approval for Sinopharm CNBG"s COVID-19 Vaccine (2020). Available at: http://www.sinopharm.com/en/s/1395-4173-38862.html (Accessed January 2, 2020).

[56] World Health Organization. Considerations for evaluation of COVID-19 vaccines (2020). Available at: https://www.who.int/medicines/regulation/prequalification/prequal-vaccines/WHO_Evaluation_Covid_Vaccine.pdf?ua=1 (Accessed September 24, 2020).

[57] Food and Drug Administration. Emergency Use Authorization for Vaccines to Prevent COVID-19-Guidance for Industry (2020). Available at: https://www.fda.gov/regulatory-information/search-fda-guidance-documents/emergency-use-authorization-vaccines-prevent-covid-19 (Accessed February 22, 2021).

[58] European Medicines Agency. EMA considerations on COVID-19 vaccine approval (2020). Available at: https://www.ema.europa.eu/en/ema-considerations-covid-19-vaccine-approval (Accessed November 19, 2020).

[59] Marshall L. Why older adults must go to the front of the vaccine line . Available at: https://medicalxpress.com/news/2021-01-older-adults-front-vaccine-line.html (Accessed January 21, 2021).

[60] Bubar KM Reinholt K Kissler SM Lipsitch M Cobey S Grad YH. Model-informed COVID-19 vaccine prioritization strategies by age and serostatus. medRxiv (2020). 10.1101/2020.09.08.20190629 PMC7963218 33479118

[61] Gan N. Chinese Covid-19 vaccine far less effective than initially claimed in Brazil, sparking concerns (2021). Available at: https://edition.cnn.com/2021/01/13/asia/sinovac-covid-vaccine-efficacy-intl-hnk/index.html (Accessed January 14, 2021).

[62] B.1.351 report. Available at: https://cov-lineages.org/global_report_B.1.351.html (Accessed Feb 15 2021).

[63] Tegally H Wilkinson E Giovanetti M Iranzadeh A Fonseca V Giandhari J. Emergence and rapid spread of a new severe acute respiratory syndrome-related coronavirus 2 (SARS-CoV-2) lineage with multiple spike mutations in South Africa. medRxiv (2020). 10.1101/2020.12.21.20248640

[64] P.1 report. Available at: https://cov-lineages.org/global_report_P.1.html (Accessed Feburary 15, 2021).

[65] Li Q Nie J Wu J Zhang L Ding R Wang H. SARS-CoV-2 501Y.V2 variants lack higher infectivity but do have immune escape. Cell (2021). 10.1016/j.cell.2021.02.042 PMC7901273 33735608

[66] Wibmer CK Ayres F Hermanus T Madzivhandila M Kgagudi P Oosthuysen B. SARS-CoV-2 501Y.V2 escapes neutralization by South African COVID-19 donor plasma. Nat Med (2021). 10.1038/s41591-021-01285-x 33654292

[67] Novavax COVID-19 vaccine demonstrates 89.3% efficacy in UK Phase 3 trial. Available at: https://ir.novavax.com/news-releases/news-release-details/novavax-covid-19-vaccine-demonstrates-893-efficacy-uk-phase-3 (Accessed Feburary 15, 2021).

[68] Johnson & Johnson announces single-shot Janssen COVID-19 vaccine candidate met primary endpoints in interim analysis of its Phase 3 ENSEMBLE trial. Available at: https://www.jnj.com/johnson-johnson-announces-single-shot-janssen-covid-19-vaccine-candidate-met-primary-endpoints-in-interim-analysis-of-its-phase-3-ensemble-trial (Accessed Feburary 15, 2021).

[69] Chen P Nirula A Heller B Gottlieb RL Boscia J Morris J. SARS-CoV-2 Neutralizing Antibody LY-CoV555 in Outpatients with Covid-19. N Engl J Med (2020) 384:229–37. 10.1056/NEJMoa2029849 PMC7646625 33113295

[70] Huang AT Garcia-Carreras B Hitchings MDT Yang B Katzelnick LC Rattigan SM. A systematic review of antibody mediated immunity to coronaviruses: kinetics, correlates of protection, and association with severity. Nat Commun (2020) 11:4704. 10.1038/s41467-020-18450-4 32943637 PMC7499300

[71] McMahan K Yu J Mercado NB Loos C Tostanoski LH Chandrashekar A. Correlates of protection against SARS-CoV-2 in rhesus macaques. Nature (2020) 590:630–4. 10.1038/s41586-020-03041-6 PMC7906955 33276369

[72] Lilly Investors. Lilly"s neutralizing antibody bamlanivimab (LY-CoV555) receives FDA emergency use authorization for the treatment of recently diagnosed COVID-19 (2020). Available at: https://investor.lilly.com/news-releases/news-release-details/lillys-neutralizing-antibody-bamlanivimab-ly-cov555-receives-fda (Accessed November 9, 2020).

[73] Lilly Investors. Lilly"s neutralizing antibody bamlanivimab (LY-CoV555) prevented COVID-19 at nursing homes in the BLAZE-2 trial, reducing risk by up to 80 percent for residents (2021). Available at: https://investor.lilly.com/news-releases/news-release-details/lillys-neutralizing-antibody-bamlanivimab-ly-cov555-prevented (Accessed January 21, 2021).

[74] Wang X Guo X Xin Q Pan Y Hu Y Li J. Neutralizing Antibodies Responses to SARS-CoV-2 in COVID-19 Inpatients and Convalescent Patients. Clin Infect Dis (2020) 71:2688–94. 10.1093/cid/ciaa721 PMC7314147 32497196

[75] Lee WT Girardin RC Dupuis AP Kulas KE Payne AF Wong SJ. Neutralizing Antibody Responses in COVID-19 Convalescent Sera. J Infect Dis (2021) 223:47–55. 10.1093/infdis/jiaa673 33104179 PMC7665673

[76] Keech C Albert G Cho I Robertson A Reed P Neal S. Phase 1-2 Trial of a SARS-CoV-2 Recombinant Spike Protein Nanoparticle Vaccine. N Engl J Med (2020) 396:887–97. 10.1056/NEJMoa2026920 PMC7494251 32877576

[77] Richmond P Hatchuel L Dong M Ma B Hu B Smolenov I. A first-in-human evaluation of the safety and immunogenicity of SCB-2019, an adjuvanted, recombinant SARS-CoV-2 trimeric S-protein subunit vaccine for COVID19 in healthy adults; a phase 1, randomised, double-blind, placebo-controlled trial. medRxiv (2020). 10.1101/2020.12.03.20243709

[78] Jackson LA Anderson EJ Rouphael NG Roberts PC Makhene M Coler RN. An mRNA Vaccine against SARS-CoV-2 - Preliminary Report. N Engl J Med (2020) 383:1920–31. 10.1056/NEJMoa2022483 PMC7377258 32663912

[79] Walsh EE Frenck RW Falsey AR Kitchin N Absalon J Gurtman A. Safety and Immunogenicity of Two RNA-Based Covid-19 Vaccine Candidates. N Engl J Med (2020) 383:2439–50. 10.1056/NEJMoa2027906 PMC7583697 33053279

[80] Xia S Zhang Y Wang Y Wang H Yang Y Gao GF. Safety and immunogenicity of an inactivated SARS-CoV-2 vaccine, BBIBP-CorV: a randomised, double-blind, placebo-controlled, phase 1/2 trial. Lancet Infect Dis (2020) 21:39–51. 10.1016/s1473-3099(20)30831-8 33069281 PMC7561304

[81] Xia S Duan K Zhang Y Zhao D Zhang H Xie Z. Effect of an Inactivated Vaccine Against SARS-CoV-2 on Safety and Immunogenicity Outcomes: Interim Analysis of 2 Randomized Clinical Trials. Jama (2020) 324:1–10. 10.1001/jama.2020.15543 PMC7426884 32789505

[82] Zhang Y Zeng G Pan H Li C Hu Y Chu K. Safety, tolerability, and immunogenicity of an inactivated SARS-CoV-2 vaccine in healthy adults aged 18-59 years: a randomised, double-blind, placebo-controlled, phase 1/2 clinical trial. Lancet Infect Dis (2020) 21:181–92. 10.1016/s1473-3099(20)30843-4 PMC7832443 33217362

[83] Zhu FC Li YH Guan XH Hou LH Wang WJ Li JX. Safety, tolerability, and immunogenicity of a recombinant adenovirus type-5 vectored COVID-19 vaccine: a dose-escalation, open-label, non-randomised, first-in-human trial. Lancet (2020) 395:1845–54. 10.1016/s0140-6736(20)31208-3 PMC7255193 32450106

[84] Yang S Li Y Dai L Wang J He P Li C. Safety and immunogenicity of a recombinant tandem-repeat dimeric RBD protein vaccine against COVID-19 in adults: pooled analysis of two randomized, double-blind, placebo-controlled, phase 1 and 2 trials. medRxiv (2020). 10.1101/2020.12.20.20248602 PMC7990482 33773111

[85] Ramasamy MN Minassian AM Ewer KJ Flaxman AL Folegatti PM Owens DR. Safety and immunogenicity of ChAdOx1 nCoV-19 vaccine administered in a prime-boost regimen in young and old adults (COV002): a single-blind, randomised, controlled, phase 2/3 trial. Lancet (2020) 396:1979–93. 10.1016/s0140-6736(20)32466-1 PMC7674972 33220855

[86] Bahl K Senn JJ Yuzhakov O Bulychev A Brito LA Hassett KJ. Preclinical and Clinical Demonstration of Immunogenicity by mRNA Vaccines against H10N8 and H7N9 Influenza Viruses. Mol Ther (2017) 25:1316–27. 10.1016/j.ymthe.2017.03.035 PMC5475249 28457665

[87] Richner JM Himansu S Dowd KA Butler SL Salazar V Fox JM. Modified mRNA Vaccines Protect against Zika Virus Infection. Cell (2017) 168:1114–25.e1110. 10.1016/j.cell.2017.02.017 28222903 PMC5388441

[88] World Health Organization. mRNA vaccines against COVID-19: Pfizer-BioNTech COVID-19 vaccine BNT162b2 (2020). Available at: https://apps.who.int/iris/handle/10665/338096 (Accessed December 22 2020).

[89] Castells MC Phillips EJ. Maintaining Safety with SARS-CoV-2 Vaccines. N Engl J Med (2020) 384:640–9. 10.1056/NEJMra2035343 PMC7787218 33378605

[90] Centers for Disease Control and Prevention. Allergic Reactions Including Anaphylaxis After Receipt of the First Dose of Pfizer-BioNTech COVID-19 Vaccine — United States, December 14–23, 2020 (2021). Centers for Disease Control. Available at: https://www.cdc.gov/mmwr/volumes/70/wr/mm7002e1.htm?s_cid=mm7002e1_x (Accessed January 15, 2021).

[91] Reichmuth AM Oberli MA Jaklenec A Langer R Blankschtein D. mRNA vaccine delivery using lipid nanoparticles. Ther Deliv (2016) 7:319–34. 10.4155/tde-2016-0006 PMC5439223 27075952

[92] Su S Du L Jiang S. Learning from the past: development of safe and effective COVID-19 vaccines. Nat Rev Microbiol (2021) 19:211–9. 10.1038/s41579-020-00462-y PMC7566580 33067570

[93] National Medical Products Administration. Guiding Principles for Clinical Evaluation of COVID-19 Preventive Vaccines (2020). Available at: http://www.cde.org.cn/zdyz.do?method=largePage&id=5b023718114d3d9d (Accessed April, 2020).

[94] Hodgson SH Mansatta K Mallett G Harris V Emary KRW Pollard AJ. What defines an efficacious COVID-19 vaccine? A review of the challenges assessing the clinical efficacy of vaccines against SARS-CoV-2. Lancet Infect Dis (2021) 21:e26–35. 10.1016/s1473-3099(20)30773-8 PMC7837315 33125914

[95] Jin P Li J Pan H Wu Y Zhu F. Immunological surrogate endpoints of COVID-2019 vaccines: the evidence we have versus the evidence we need. Signal Transduct Target Ther (2021) 6:48. 10.1038/s41392-021-00481-y 33531462 PMC7851657

[96] World Health Organization. Main outcomes of the meeting of the WHO Expert Committee on Biological Standardization held from 24 to 28 August 2020. WHO website (2020). Available at: https://www.who.int/docs/default-source/biologicals/ecbs/who-ecbs-august-2020-executive-summary-final-ik-if-tw-1-sep-2020.pdf?sfvrsn=4fef7033_8.

[97] National Institute for Biological Standards and Control. First WHO International Standard Anti-SARS-CoV-2 Immunoglobulin (Human). NIBSC website (2020). Available at: https://www.nibsc.org/products/brm_product_catalogue/detail_page.aspx?catid=20/136.

[98] National Institute for Biological Standards and Control. NIBSC First WHO International Standard for SARS-CoV-2 RNA (2020).

[99] World Health Organization. Standardization of vaccines for coronavirus disease (COVID-19) (2021). Available at: https://www.who.int/news-room/feature-stories/detail/standardization-of-vaccines-for-coronavirus-disease-covid-19 (Accessed January 12, 2021).

[100] Kim JH Excler JL Michael NL. Lessons from the RV144 Thai phase III HIV-1 vaccine trial and the search for correlates of protection. Annu Rev Med (2015) 66:423–37. 10.1146/annurev-med-052912-123749 25341006

[101] Excler JL Kim JH. Novel prime-boost vaccine strategies against HIV-1. Expert Rev Vaccines (2019) 18:765–79. 10.1080/14760584.2019.1640117 31271322

[102] Zhang L Jackson CB Mou H Ojha A Rangarajan ES Izard T. The D614G mutation in the SARS-CoV-2 spike protein reduces S1 shedding and increases infectivity. bioRxiv (2020) 2020.2006.2012.148726. 10.1101/2020.06.12.148726

[103] Davies NG Barnard RC Jarvis CI Kucharski AJ Munday JD Pearson CAB. Estimated transmissibility and severity of novel SARS-CoV-2 Variant of Concern 202012/01 in England. bioRxiv (2020). 10.1101/2020.12.24.20248822

[104] Wibmer CK Ayres F Hermanus T Madzivhandila M Kgagudi P Lambson BE. SARS-CoV-2 501Y.V2 escapes neutralization by South African COVID-19 donor plasma. bioRxiv (2021). 10.1101/2021.01.18.427166 33654292

[105] Cele S Gazy I Jackson L Hwa S-H Tegally H Lustig G. Escape of SARS-CoV-2 501Y.V2 variants from neutralization by convalescent plasma. medRxiv (2021). 10.1101/2021.01.26.21250224 PMC9867906 33780970

[106] Shen X Tang H McDana C Wagh K Fischer W Theiler J. SARS-CoV-2 variant B.1.1.7 is susceptible to neutralizing antibodies elicited by ancestral Spike vaccines. medRxiv (2021). 10.1101/2021.01.27.428516 PMC7934674 33705729

[107] Wang P Liu L Iketani S Luo Y Guo Y Wang M. Increased Resistance of SARS-CoV-2 Variants B.1.351 and B.1.1.7 to Antibody Neutralization. bioRxiv (2021). 10.1101/2021.01.25.428137 33684923

[108] Collier D Meng B Ferreira I Datir R Temperton N Elmer A. Impact of SARS-CoV-2 B.1.1.7 Spike variant on neutralisation potency of sera from individuals vaccinated with Pfizer vaccine BNT162b2. medRxiv (2021). 10.1101/2021.01.19.21249840

[109] Wu K Werner AP Moliva JI Koch M Choi A Stewart-Jones GBE. mRNA-1273 vaccine induces neutralizing antibodies against spike mutants from global SARS-CoV-2 variants. bioRxiv (2021). 10.1101/2021.01.25.427948

[110] Ong E Wong MU Huffman A He Y. COVID-19 Coronavirus Vaccine Design Using Reverse Vaccinology and Machine Learning. Front Immunol (2020) 11:1581. 10.3389/fimmu.2020.01581 32719684 PMC7350702

[111] Ong E Huang X Pearce R Zhang Y He Y. Computational design of SARS-CoV-2 spike glycoproteins to increase immunogenicity by T cell epitope engineering. Comput Struct Biotechnol J (2021) 19:518–29. 10.1016/j.csbj.2020.12.039 PMC7773544 33398234

[112] Oyarzun P Kashyap M Fica V Salas-Burgos A Gonzalez-Galarza FF McCabe A. A Proteome-Wide Immunoinformatics Tool to Accelerate T-Cell Epitope Discovery and Vaccine Design in the Context of Emerging Infectious Diseases: An Ethnicity-Oriented Approach. Front Immunol (2021) 12:598778. 10.3389/fimmu.2021.598778 33717077 PMC7952308

[113] Gao Q Bao L Mao H Wang L Xu K Yang M. Development of an inactivated vaccine candidate for SARS-CoV-2. Science (2020) 369:77–81. 10.1126/science.abc1932 32376603 PMC7202686

[114] Wang H Zhang Y Huang B Deng W Quan Y Wang W. Development of an Inactivated Vaccine Candidate, BBIBP-CorV, with Potent Protection against SARS-CoV-2. Cell (2020) 182:713–21.e719. 10.1016/j.cell.2020.06.008.s 32778225 PMC7275151

